# Does the Position or Contact Pressure of the Stethoscope Make Any Difference to Clinical Blood Pressure Measurements: An Observational Study

**DOI:** 10.1097/MD.0000000000000301

**Published:** 2014-12-02

**Authors:** Fan Pan, Dingchang Zheng, Peiyu He, Alan Murray

**Affiliations:** From the College of Electronics and Information Engineering, Sichuan University, Chengdu, Sichuan 610065, P.R. China (FP, PH); and Cardiovascular Physics and Engineering Research Group, Institute of Cellular Medicine, Newcastle University, Newcastle upon Tyne NE2 4HH, UK (FP, DZ, AM).

## Abstract

This study aimed to investigate the effect of stethoscope position and contact pressure on auscultatory blood pressure (BP) measurement.

Thirty healthy subjects were studied. Two identical stethoscopes (one under the cuff, the other outside the cuff) were used to simultaneously and digitally record 2 channels of Korotkoff sounds during linear cuff pressure deflation. For each subject, 3 measurements with different contact pressures (0, 50, and 100 mm Hg) on the stethoscope outside the cuff were each recorded at 3 repeat sessions. The Korotkoff sounds were replayed twice on separate days to each of 2 experienced listeners to determine systolic and diastolic BPs (SBP and DBP). Variance analysis was performed to study the measurement repeatability and the effect of stethoscope position and contact pressure on BPs.

There was no significant BP difference between the 3 repeat sessions, between the 2 determinations from each listener, between the 2 listeners and between the 3 stethoscope contact pressures (all *P* > 0.06). There was no significant SBP difference between the 2 stethoscope positions at the 2 lower stethoscope pressures (*P* = 0.23 and 0.45), but there was a small (0.4 mm Hg, clinically unimportant) significant difference (*P* = 0.005) at the highest stethoscope pressure. The key result was that, DBP from the stethoscope under the cuff was significantly lower than that from outside the cuff by 2.8 mm Hg (*P* < 0.001, 95% confidence interval −3.5 to −2.1 mm Hg).

Since it is known that the traditional Korotkoff sound method, with the stethoscope outside the cuff, tends to give a higher DBP than the true intra-arterial pressure, this study could suggest that the stethoscope position under the cuff, and closer to the arterial occlusion, might yield measurements closer to the actual invasive DBP.

## INTRODUCTION

Blood pressure (BP) is commonly measured non-invasively by manual auscultatory and automatic oscillometric methods. The manual auscultatory method is the gold standard for clinical BP measurement, and requires a cuff, a stethoscope and a cuff pressure display.^1^ A trained clinician or nurse uses the stethoscope to listen for the Korotkoff sounds associated with blood flowing through the brachial arm artery as a BP cuff encircling the upper arm is deflated.^2^ The appearance and disappearance of sounds is associated with systolic and diastolic blood pressures (SBP and DBP) respectively, and the BPs at these times are read from a cuff pressure display.

However, manual auscultation is often inaccurately performed. It has been reported that some of the inaccuracies are associated with measurement conditions, including patient posture, arm position, back support, cuff size, cuff pressure deflation rate, and the environment in which BP measurements are made.^3–7^ Various international societies of hypertension, including the American Heart Association, the European Society of Hypertension and the British Hypertension Society^6,8,9^ have produced guidelines to carefully control the conditions for achieving accurate and reliable BP measurement in humans. It has been recommended that the stethoscope should be placed gently over the brachial artery at the point of maximal pulsation, and held firmly and evenly but without excessive pressure.^1,6^ In clinical practice, the stethoscope is sometimes placed under the cuff, but traditionally and more usually outside the cuff over the brachial artery in the antecubital fossa. Placing the stethoscope at the 2 positions may generate different Korotkoff sounds, resulting in different interpretation by observers and hence measurement errors in BP determination. However there is little scientific evidence to quantify the BP difference between the measurements taken with the stethoscope under the cuff and outside the cuff. Also, when clinicians hold the stethoscope outside the cuff during the measurement, there is no specific recommendation of how much contact pressure the operators should apply on the stethoscope, and hence no evidence of its effect on BP determination.

The importance of accurate BP measurement in clinical practice is without doubt, and small inaccuracies in BP measurement can have considerable consequences.^10^ It has been reported by population studies that overestimating or underestimating BP by even 5 mm Hg can seriously compromise diagnosis, resulting in millions of people being wrongly diagnosed as hypertensive with attendant exposure to adverse drug effects, or being denied treatment leading to associated cardiovascular conditions, including fatal stroke and fatal myocardial infarction.^11,12^ Therefore, any potential small BP differences caused by the stethoscope position or the contact pressure applied on the stethoscope head are clinically important, and worth further investigation.

The aim of this study was to investigate the effect on auscultatory BP measurement of the stethoscope position and contact pressure on the stethoscope head.

## METHODS

### Subjects

A sample size calculation was performed based on a paired *t* test for mean difference. The required sample size was estimated allowing for a mean 5 mm Hg BP difference, which is considered to be clinically significant, to be detected with a typical 8 mm Hg standard deviation (SD) of BP measurement; 21 subjects were required to achieve a confidence level of 95% with a statistical power of 80%. Thirty healthy subjects (13 male and 17 female) were recruited from April to May 2013, with ages from 23 to 63 years. There were 9, 10, and 11 subjects within the age bands of 20 to 29, 30 to 39, and 40 years and over, respectively. They were mainly from the staff, students and visitors of Freeman Hospital and Newcastle University.

Exclusion criteria for this study included subjects aged under 18 years or over 80 years; subjects with known cardiovascular disease including the atrial fibrillation or other irregular heart rhythms; and subjects who were pregnant.

This study received ethical permission from the Newcastle & North Tyneside Research Ethics Committee. The investigation conformed with the principles in the Declaration of Helsinki. None of the subjects had any known cardiovascular disease. All subjects gave their written informed consent to participate in the study. Table 1 briefly summarizes the subject demographic information, including age, sex, height, weight, and arm circumference.

**TABLE 1:**
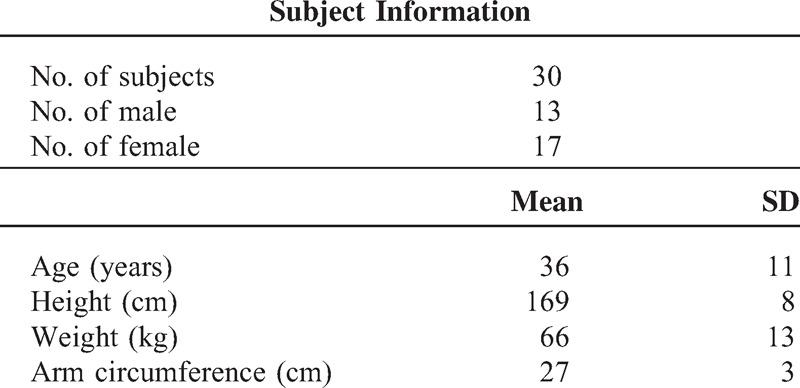
General Data Information for the Subjects Studied

### BP Measurement

All BP measurements were performed in a quiet and temperature-controlled clinical measurement room by a trained operator at the Freeman Hospital, Newcastle upon Tyne, UK. Prior to the formal recording, the subject was asked to rest on a chair for 5 minutes. They were also asked to breathe gently during the measurement. The whole procedure followed the guidelines recommended by the British Hypertension Society and American Heart Association.^1,8^

**FIGURE 1:**
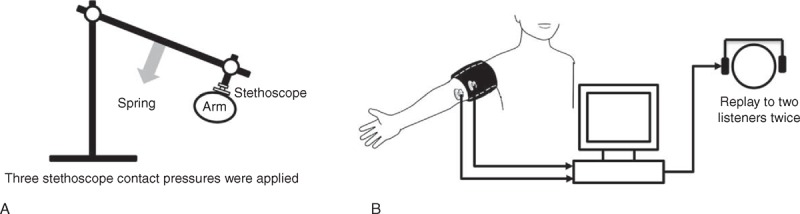
(A) Diagram of the holder used to apply different stethoscope contact pressures, and (B) measurement system for Korotkoff sound recording.

**FIGURE 2:**
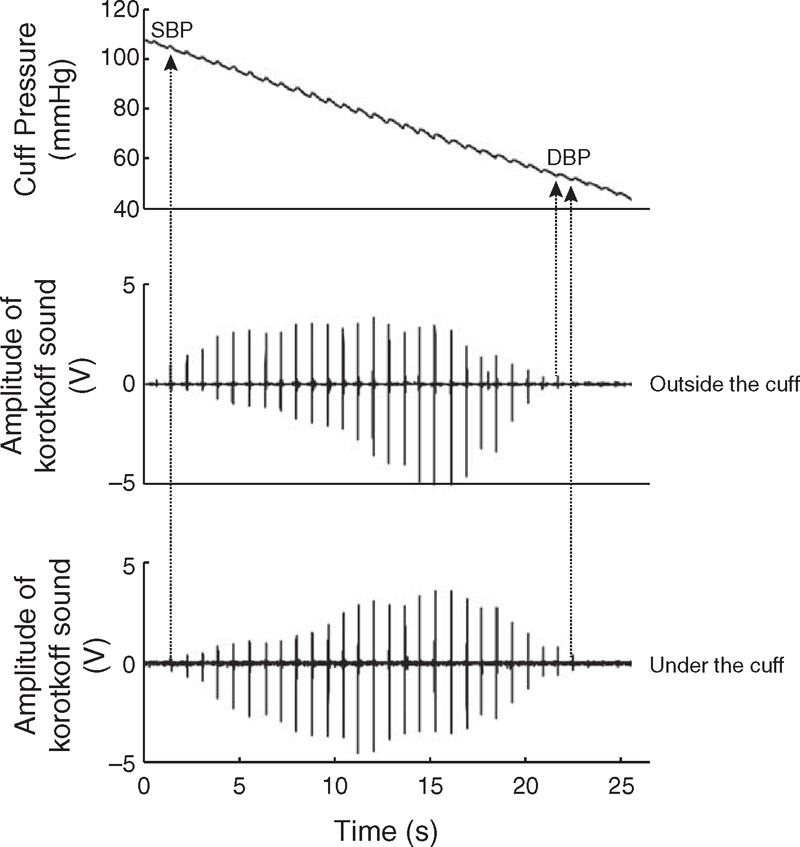
Example of Korotkoff sounds recorded with the stethoscope outside (middle trace) and under (bottom trace) the cuff. The sounds associated with systolic blood pressure (SBP) and diastolic blood pressure (DBP) are identified. The scales of Korotkoff sounds have arbitrary, but consistent, units.

As shown in Figure 1, 2 identical stethoscopes were used with one placed under the cuff and the other outside the cuff on the antecubital fossa. A specially designed holder with a spring scale was used to position the stethoscope outside the cuff and then apply 3 different levels of skin contact pressure (0, 50, 100 mm Hg) on this stethoscope head. During cuff deflation, 1 channel of cuff pressure and 2 channels of Korotkoff sounds were simultaneously and digitally recorded to a data capture computer at a sample rate of 2000 Hz. Cuff pressure was linearly deflated at a standard rate of 2 to 3 mm Hg/s. Figure 2 illustrates a typical example of recorded cuff pressure and Korotkoff sounds from both stethoscopes.

For each subject, there were 3 repeated sessions with 3 measurements for each, giving a total of 9 recordings. A time interval of at least 4 minutes was given between sessions, and at least 1 minute between the 3 measurements within a session, allowing recovery of cardiovascular hemodynamics. For the 3 measurements within a session, 3 different stethoscope contact pressures (0, 50, and 100 mm Hg) were applied sequentially on the stethoscope outside the cuff, with the sequence of these different levels of contact pressure randomised between subjects.

### BP Determination

For each subject, 9 cuff pressure signals and 18 recordings of Korotkoff sound (from 3 repeat recordings of 3 contact pressures, and 2 stethoscope positions) were analysed off-line. Software developed using Matlab 2011a (MathWork Inc, Massachusetts, USA) was used to convert the recorded Korotkoff sound signals into. wav files. Because there are potential BP measurement bias from different listeners and repeat determinations,^13–15^ all the sound recordings were replayed twice (on 2 different days) to 2 trained listeners. The replay order of all 540 recorded Korotkoff sounds (from 18 Korotkoff sounds for each subject × 30 subjects) was randomised, and the listeners were unaware of any subject or stethoscope information. Figure 2 presents an example of SBP and DBP determination. The listener identified the appearance and disappearance of the sounds by clicking the computer mouse. The baseline cuff pressure at which the Korotkoff sound appears is associated with SBP, and the cuff pressure at which the Korotkoff sound disappears is associated with DBP.

### Data and Statistical Analysis

In total, there were 72 values from each subject (from 2 stethoscope positions, 3 contact pressures, 3 repeat recordings, 2 listeners and 2 BP determinations on separate days by each listener) for both SBP and DBP. The overall mean and SD of the BPs were calculated across all subjects, separately for the 2 stethoscope positions, 3 contact pressures and 2 listeners.

The SPSS Statistics 19 software package (SPSS Inc, Chicago, IL, USA) was employed to perform analysis of variance (ANOVA) analysis to study the measurement repeatability between the 3 repeat measurement sessions, between the 2 listeners, between the 2 determinations from each listener, and the effect of stethoscope position and contact pressure. The mean BP differences between the above factors were also analysed, and the histograms of BP differences plotted. All differences were paired values in each subject, and a value of *P* < 0.05 was considered a statistically significant difference. Finally, linear regression analysis was performed to investigate the relationship between age and BP differences between the measurements taken from the stethoscope under and outside the cuff.

## RESULTS

### BP Repeatability

**FIGURE 3:**
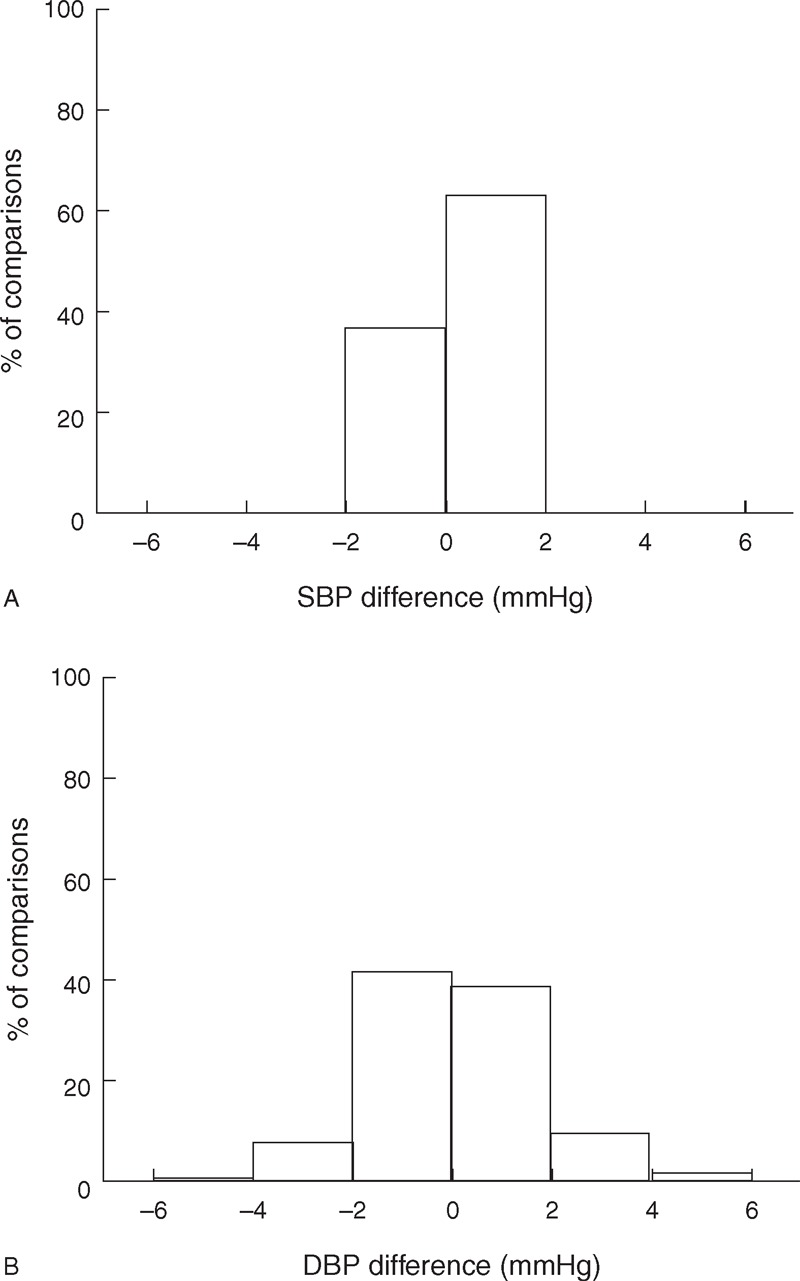
Histogram of within-subject systolic blood pressure (SBP) (A) and diastolic blood pressure (DBP) (B) differences between the 2 listeners. For each sound recording, the measurements from the 2 listeners were compared. A total of 1080 comparisons (from 30 subjects, 2 stethoscope positions, 3 contact pressures, 3 repeat recording sessions and 2 BP determinations on separate days) were made.

Statistical analysis showed that there was no significant BP differences (for both SBP and DBP) between the 3 repeat measurement sessions, between the 2 determinations from each listener and between the 2 listeners (all *P* > 0.06). As shown in Figure 3, over 95% of SBP and over 80% of DBP measurements had a difference of no more than 2 mm Hg between the 2 listeners.

### Effect of Stethoscope Contact Pressure and Position on BP

Variance analysis showed that the effect of stethoscope contact pressure on both SBP and DBP was not statistically significant (*P* *=* 0.07 for SBP and *P* *=* 0.10 for DBP), indicating that different contact pressures on the stethoscope outside the cuff did not influence BP determination.

**FIGURE 4:**
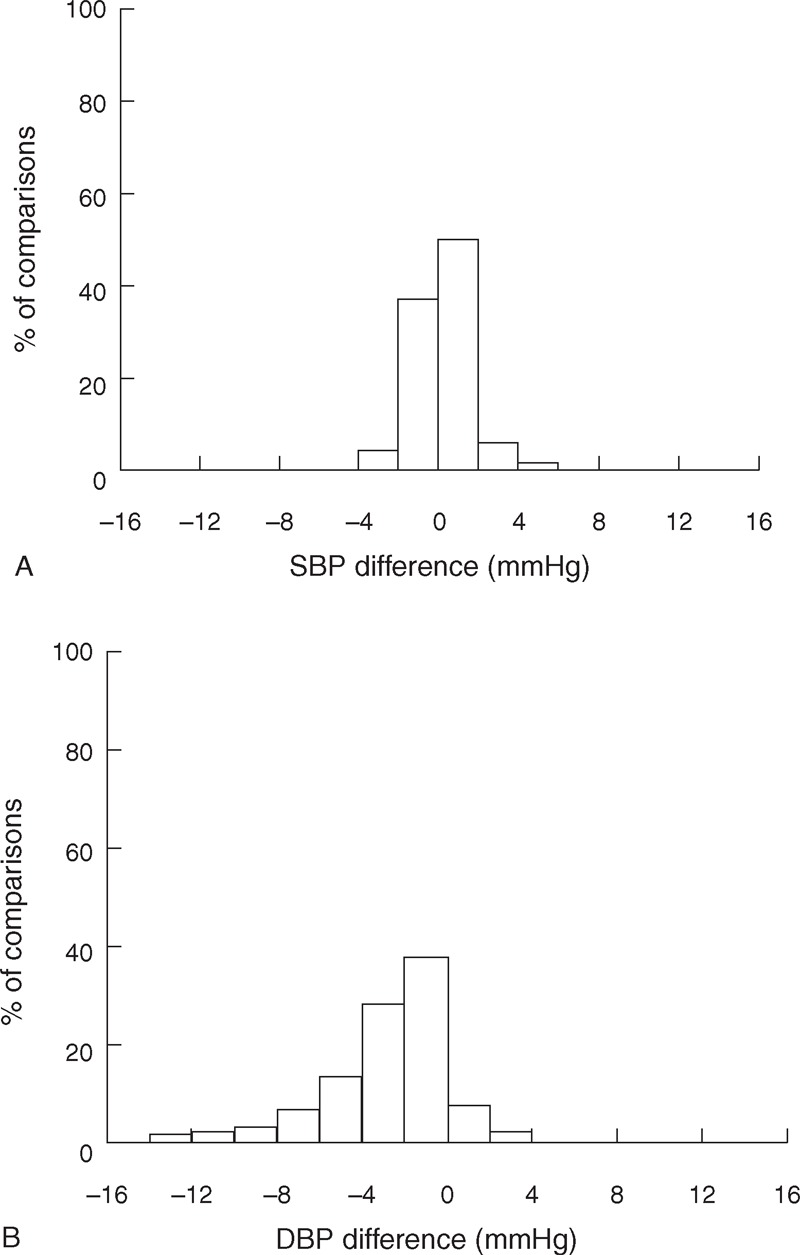
Histogram of within-subject systolic blood pressure (SBP) (A) and diastolic blood pressure (DBP) (B) differences between the measurements taken under the cuff and outside the cuff. A total of 1080 comparisons (from 30 subjects, 3 contact pressures, 3 repeat recording sessions, 2 listeners and 2 BP determinations on separate days) were used.

**FIGURE 5:**
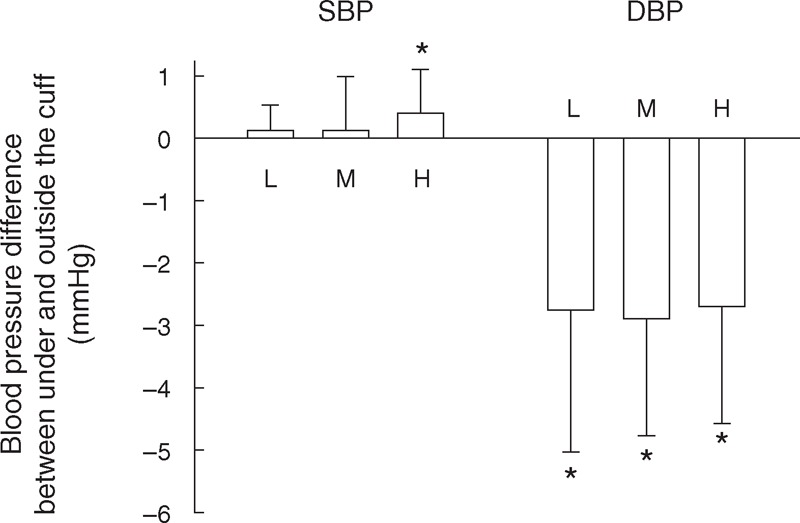
Overall mean and SD of within-subject systolic blood pressure (SBP) and diastolic blood pressure (DBP) differences between the measurements taken from the stethoscopes under and outside the cuff. Differences at 3 contact pressures on the outside stethoscope head (L: 0 mm Hg, M: 50 mm Hg, H: 100 mm Hg) are given. ^∗^Significant difference, *P* < 0.05.

However, there were small BP differences between the 2 stethoscope positions. As shown in Figure 4, 13% of SBP measurements and 55% of DBP measurements had a difference of more than 2 mm Hg. More specifically, for SBP, at the highest level of stethoscope contact pressure (100 mm Hg), SBP from the stethoscope under the cuff pressure was 0.4 mm Hg statistically significantly higher than that from outside the cuff (*P* *=* 0.005, 95% confidence interval 0.1–0.7 mm Hg), which is shown in Figure 5 and Table 2.

**TABLE 2:**
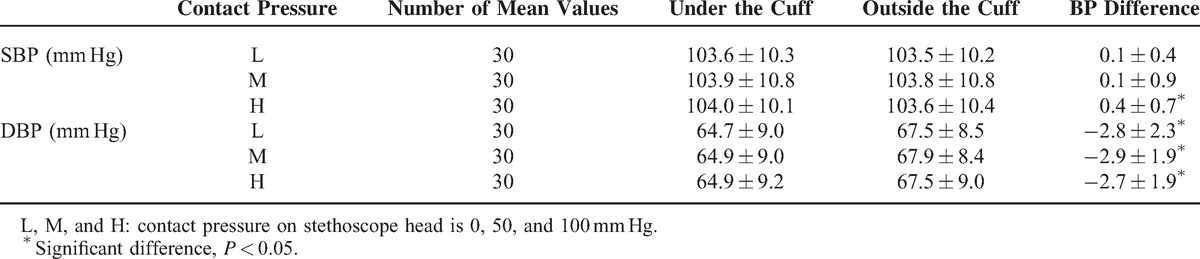
Overall Blood Pressures Measured With Stethoscope Under and Outside the Cuff From 30 Subjects. Their Within-Subject Differences Are Also Given. For Each Subject, the Average BPs From 3 Repeat Recording Sessions, 2 Listeners and 2 BP Determinations on Separate Days Were Used

For DBP, there were statistically significant differences between the 2 stethoscope positions at all 3 stethoscope contact pressures (all *P* < 0.001). Overall, DBP from the stethoscope under the cuff was statistically significantly lower by 2.8 mm Hg (95% confidence interval −3.5 to −2.1 mm Hg) than that from outside the cuff (*P* < 0.001).

### Effect of Age on BP Difference

There was no significant relationship between age and BP differences between the measurements taken under and outside the cuffs (*P* > 0.7 for both SBP and DBP differences).

## DISCUSSION

Our study has quantitatively shown that DBPs measured with the stethoscope under and outside the cuff were different. To the best of our knowledge, this is the first clinical study to simultaneously compare the BP difference from different stethoscope positions. Considering the cuff pressure deflation rate of 2 to 3 mm Hg/s, an overall 2.8 mm Hg DBP difference suggests that there is on average approximately 1 beat difference between the measurements from the stethoscope under and outside the cuff. This DBP measurement difference, affected by stethoscope position, provides further evidence that it is difficult to measure DBP accurately.^8^ It also has implications for actual DBP measurement. It is known that the traditional Korotkoff sound method, with the stethoscope outside the cuff, tends to give a higher DBP than the true intra-arterial pressure, which Nielsen et al have shown to be different by 6 mm Hg (see Table 5 of Nielsen et al's study).^16^ Our study has provided quantitative evidence and confirmed that manual DBP measurement from the stethoscope under the cuff could achieve closer values to the true invasive measurement.

One possible explanation for the DBP difference is that, during cuff pressure deflation, the effect of arterial flow is heard more easily under the cuff, resulting in the diastolic Korotkoff sounds from the stethoscope outside the cuff disappearing before these from under the cuff, which is shown in Figure 2. It can also be seen in Figure 2 that the envelopes of Korotkoff sound amplitudes are different between the 2 simultaneously recorded signals, which may also suggest different mechanical behaviour of the brachial arteries at the 2 positions during BP measurement.^17^ In addition, the stethoscope head might induce a deformity in the brachial artery that could disturb blood flow, potentially influencing DBP. It would therefore be useful to understand the blood flow during BP measurement using a Doppler device.

However, a significant difference was not observed for SBP between the 2 positions when the stethoscope outside the cuff was applied with low and medium contact pressures. Even for the comparison at the high stethoscope contact pressure (100 mm Hg), the difference was only 0.4 mm Hg. It is noted that this mean SBP difference of 0.4 mm Hg cannot be considered to be clinically significant.

Another finding of this study is that different contact pressures (up to 100 mm Hg) applied locally on the stethoscope head does not influence manual ausculatory BP measurement. Our results partially agreed with Londe's study,^18^ where they concluded that excessive pressure on the stethoscope head in auscultatory BP measurement does not affect SBP. However, significantly lower DBP readings were observed with a stethoscope contact pressure of 100 mm Hg in their study. It could be caused by the different way they introduced the stethoscope contact pressure. They applied contact pressure through a 9-cm cuff wrapped around the forearm over the stethoscope, however, in our study the pressure was applied locally on the stethoscope head, which is closer to the real clinical measurement situation. In order to provide comfort for subjects being measured and avoid distorting the artery, placing the stethoscope gently and evenly but without excessive pressure over the brachial artery should be followed as recommended the BP measurement guidelines.^1,8^

One limitation of our study is that the BPs were obtained in this study by clicking the computer mouse after determining the appearance and disappearance of the Korotkoff sounds, which would generate some delays in BP determinations. However, since all measurements were made in the same way, and since BP difference was being investigated, such delays would cancel. Secondly, although we used a standard stethoscope head and recorded the sounds using a microphone with better frequency characteristics than the human ear, a clinical study should be performed to validate the measurement technique using an electronic microphone against the direct manual auscultatory method in a clinical setting. Thirdly, the total population sample of 30 healthy subjects would need to be expanded in the future to study the effect of age and hypertension.

In summary, the effect of stethoscope position and stethoscope contact pressure has been quantified, providing scientific evidence that the stethoscope position is one of the factors influencing BP measurement. This study could also suggest that the stethoscope position under the cuff might yield measurements closer to the actual invasive DBP.
